# Retraction Note to: Gene expression analysis indicates CB1 receptor upregulation in the hippocampus and neurotoxic effects in the frontal cortex 3 weeks after single-dose MDMA administration in Dark Agouti rats

**DOI:** 10.1186/s12864-016-3072-9

**Published:** 2016-09-08

**Authors:** Peter Petschner, Viola Tamasi, Csaba Adori, Eszter Kirilly, Romeo D. Ando, Laszlo Tothfalusi, Gyorgy Bagdy

**Affiliations:** 1 Department of Pharmacodynamics, Semmelweis University, H-1089 Nagyvarad ter 4., Budapest, Hungary; 2 Department of Genetics, Cell- and Immunobiology, Semmelweis University, H-1089 Nagyvarad ter 4., Budapest, Hungary; 3 MTA-SE Neuropsychopharmacology and Neurochemistry Research Group, Budapest, Hungary

## Retraction note

“This article [1] has been retracted by the authors after they discovered an honest error in the data. The error influences the fold change values of the differentially expressed genes described in the article. Through the automated process of the analysis the authors mistakenly failed to identify that these values were inverse values, and thus, the direction of changes in the individual gene analysis is opposite to those reported in the article. As a result, large sections of the article are inaccurate. However, the gene set enrichment analysis and figures 1,2,4,6 and 7 are correct in the publication. To avoid confusion this article has been retracted and the authors have been given the opportunity to resubmit their corrected data.”
